# Nitric Oxide Signaling and Its Association with Ubiquitin-Mediated Proteasomal Degradation in Plants

**DOI:** 10.3390/ijms23031657

**Published:** 2022-01-31

**Authors:** Anjali Pande, Bong-Gyu Mun, Murtaza Khan, Waqas Rahim, Da-Sol Lee, Geun-Mo Lee, Tiba Nazar Ibrahim Al Azawi, Adil Hussain, Byung-Wook Yun

**Affiliations:** 1 Laboratory of Plant Molecular Pathology and Functional Genomics, Department of Plant Biosciences, School of Applied Biosciences, College of Agriculture & Life Science, Kyungpook National University, Daegu 41944, Korea; mun0301@naver.com (B.-G.M.); murtazakhan.bio@gmail.com (M.K.); waqasrahim999@yahoo.com (W.R.); giftanna@naver.com (D.-S.L.); looxia@naver.com (G.-M.L.); redflower660@yahoo.com (T.N.I.A.A.); 2 Laboratory of Cell Biology, Department of Entomology, Abdul Wali Khan University, Mardan 23200, Khyber Pakhtunkhwa, Pakistan; adilhussain@awkum.edu.pk

**Keywords:** nitric oxide, ubiquitylation, S-nitrosylation, proteasome, proteolysis

## Abstract

Nitric oxide (NO) is a versatile signaling molecule with diverse roles in plant biology. The NO-mediated signaling mechanism includes post-translational modifications (PTMs) of target proteins. There exists a close link between NO-mediated PTMs and the proteasomal degradation of proteins via ubiquitylation. In some cases, ubiquitin-mediated proteasomal degradation of target proteins is followed by an NO-mediated post-translational modification on them, while in other cases NO-mediated PTMs can regulate the ubiquitylation of the components of ubiquitin-mediated proteasomal machinery for promoting their activity. Another pathway that links NO signaling with the ubiquitin-mediated degradation of proteins is the N-degron pathway. Overall, these mechanisms reflect an important mechanism of NO signal perception and transduction that reflect a close association of NO signaling with proteasomal degradation via ubiquitylation. Therefore, this review provides insight into those pathways that link NO-PTMs with ubiquitylation.

## 1. Introduction

Nitric oxide (NO) is a highly reactive and unstable gas with a short half-life. However, it is one of the most versatile signaling molecules in the biological system. The hunt for the presence of nitric oxide in plants began in the year 1998 [1,2], soon after its identification in the mammalian system in the year 1987 [3]. In plants, it is involved in various growth, developmental [4,5], and environmental stress acclimation processes [1,2,6]. In addition, NO is also involved in the signaling pathways that lead to programed cell death [7,8,9,10]. The main mechanism of action of NO in plants is through the post-translational modifications of proteins that are involved in cell signaling under physiological and stress conditions [11,12,13]. The NO-mediated PTMs predominantly comprise of S-nitrosylation, tyrosine nitration, and metal nitrosylation of target proteins. However, targeted proteolysis of group VII Ethylene Response Factors (ERFs-VII) transcription factor (TFs) via the N-end rule pathway [14,15] is suggested to be a general homeostatic mechanism of NO sensing in plants. This pathway was recently named as the N-degron pathway of protein degradation [16].

Ubiquitylation is a major pathway regulating proteolysis in eukaryotes [17,18]. In plants, it regulates several developmental processes indicating its diverse role throughout the life cycle of plants. Moreover, it regulates many signal transduction pathways, mostly those related to a plant’s response under environmental stress, by rapidly enhancing or degrading the proteins through the 26S proteasome, depending upon their requirements during the stress response [19]. In this pathway, multiple ubiquitin proteins are conjugated to the target protein as labels or tags (a process called ubiquitylation). The tagged proteins are directed to the 26S proteasome for degradation [19,20,21,22]. However, it is interesting to note that plants possess other degradation systems similar to ubiquitylation, such as autophagy and SUMOylation, that involve ubiquitin-like modifiers, namely ATG8 (autophagy-related protein 8) and small ubiquitin like-modifier (SUMO), respectively. Just as ubiquitin marks the proteins for degradation in the process of ubiquitylation, similarly, ATG8 surrounds the autophagosome (a double-membrane vesicle) during autophagy. Intriguingly, ubiquitin also serves in many selective autophagy processes, thus acting as a common factor for the Ubiquitin Proteasome System (UPS) and autophagy. Moreover, autophagy can also degrade components of UPS such as the 26S proteasome, while UPS can degrade the autophagosome markers, the ATGs [23].

Like Ubiquitylation, SUMOylation also involves the covalent attachment of small ubiquitin-like modifier (SUMO) to the lysine residue on the target protein. Although ubiquitin marks the target proteins for degradation, SUMOylation changes the fate of target proteins by altering their stability or interaction with other partners (proteins or DNA) [24]. However, there exists a point of interaction between SUMOylation and ubiquitylation in multiple pathways, as suggested by Denuc and Marfany [25], which is supportive of their convergent evolution in the plant system [19,26].

Although NO plays regulatory roles in autophagy [27,28,29] and SUMOylation [11,30,31], this review mainly focuses on the NO-mediated signaling and its association with ubiquitylation. Therefore, in this review, we have discussed the relationship between nitric oxide sensing via NO-mediated PTMs of target proteins and the N-degron pathway, which are associated with ubiquitin-mediated proteasomal degradation. These mechanisms highlight a valuable role of NO signal perception and transduction in plants.

## 2. An Overview of the Mechanism of Ubiquitylation in Plants

Ubiquitin-mediated proteasomal degradation or ubiquitylation is a post-translational modification involving the addition of multiple (a chain of four or more) ubiquitin tags to the target protein, and thereafter directing it to 26S proteasome for degradation. The ligation of ubiquitin on the lysine residue of the target protein (monoubiquitylation) is carried out by 3 enzymes, namely ubiquitin-activating enzymes (E1), ubiquitin-conjugating enzymes (E2) and ubiquitin ligases (E3) [21]. The adenylation of the C-terminus of ubiquitin is catalyzed by E1 which allows it to form a thioester bond between the active site cysteine of E1 and the carboxylate group of ubiquitin’s C-terminus [32]. Thereafter, the activated ubiquitin gets transferred to a cysteine residue of an E2 enzyme through a thioester bond. Finally, the covalent attachment of ubiquitin (together with the E2 enzyme) to the substrate proteins takes place through the activity of substrate-specific E3 ligase [21]. In flowering plants, there are more than one enzyme that perform the function of E1, E2 or E3 enzymes. For instance, Arabidopsis ubiquitin-activating enzymes, UBA1 and UBA2, carry out the function of E1 enzymes [33], and there is prediction of 37 E2 enzymes encoded in the genome [34]. Interestingly, the identification of around 1500 gene encoding components of E3 ligases in Arabidopsis depicts the vast substrate specificity and the significance of the ubiquitin system in plants as compared to other eukaryotes [35]. Moreover, the activity of a fourth enzyme, E4 ligase may be involved in the elongation of the polyubiquitin chains [36]. In Arabidopsis, the identification of an E4 ligase, MUTANT, SNC1-Enhancing (MUSE3) was found to be involved in the polyubiquitination of immune receptors, NLRs (proteins with nucleotide binding and leucine-rich repeat domains) [37].

Once the ubiquitin tags are attached to the substrate protein, they are targeted to the 26S proteasome for proteolysis [38]. The 26S proteasome is found in all eukaryotic cells and is located in the cytosol and nucleus as a 2.5 MDa, multi-subunit protease [39,40,41,42]. It is composed of 2 separately stable sub-complexes, the 20S core protease (CP) and the 19S regulatory particle (RP). RP is the outer cap-like structure on one or both ends, involved in capturing and preparing the appropriate substrate for degradation, while CP is a barrel shaped structure involved in peptidase activities [43,44,45]. The polyubiquitylated conjugate can either be broken down by the 26S proteasome or disassembled by the hydrolytic activity of deubiquitinating enzymes (DUBs) releasing the bound ubiquitin moieties for further use. Recent studies suggest that a variety of cellular processes in plants are regulated by DUBs in plants, such as the regulation of plant immunity [46] and in jasmonic acid signaling [47].

## 3. NO-Mediated Ubiquitylation Process in Plant Development and Stress Management

The fact that NO is a key signaling molecule involved in a plethora of physiological processes regulating plant growth, development and stress acclimation suggests that NO signal perception, transduction, and response regulation is rather more complex than studied so far. The regulation of processes like ubiquitylation indicates the active involvement of NO in maintaining the cellular levels of proteins, also known as “cellular proteostasis”, participating in various growth, development and stress acclimation pathways. In this context, the first identified NO sensing mediated by the N-degron pathway relates the regulation of the in vivo half-life of a protein based on the N-terminal residue [16]. The substrates of this pathway, such as the ETHYLENE RESPONSE FACTOR VII (ERF-VII) family of transcription factors [48], the Polycomb Repressor Complex 2 component VERNALIZATION (VRN) 2 [49] and LITTLE ZIPPER 2 (ZPR2) transcription factor [50], are destabilized by this pathway, thus regulating seed germination [51], shoot and leaf development [52], gametophyte development [53], enhanced hypoxia tolerance ([48], enhanced tolerance to abiotic stress such as drought and salt [54], recovery after dark, submergence and starvation [55], and various pathogen responses [56]. Furthermore, the enzymatic components of the N-degron pathway are also involved in seed ripening, lipid breakdown, hormone signaling and germination [57]. Though the N-degron pathway explains the link between NO sensing and proteolytic regulation to control physiological processes in plants, many associated substrates and components of different pathways associated with them still need to be identified to fully appreciate this versatile pathway in plant.

Furthermore, the expanding literature on NO sensing through NO-mediated PTMs suggests another level of control by NO in regulating the physiological responses in plants. The delicate levels of phytohormones like auxins, abscisic acid and salicylic acid involved in various growth and developmental processes and also abiotic and biotic stress responses are regulated by S-nitrosylation. A few components involved in phytohormonal synthesis, conjugation and signaling undergo ubiquitylation, thus mediating a response regulation in plants. For instance, ABI5, PYR1, TIR1, APX1 and NPR1 (discussed later in the review) are all part of the phytohormonal signaling.

## 4. Ubiquitylation as Part of NO Sensing in Plants: The N-Degron Pathway

**Figure 1:**
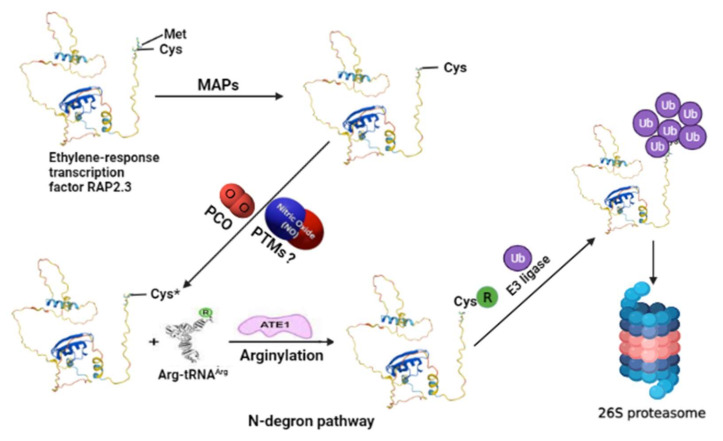
An exemplified model for understanding the N-degron pathway involving ubiquitin proteasomal degradation of ERF-VII (for example, RELATED TO AP2 3, RAP2.3) TF as a part of the NO sensing mechanism in plants. The N-terminal methionine is cleaved by MAPs exposing the second residue, cysteine (Cys). Cysteine gets oxidized by the action of PCOs. In this step, the role of NO is also reported, so possible NO-mediated oxidation of cysteine residue via PTMs still needs to be explored. The oxidized cysteine (Cys*) of the target protein then undergoes arginylation by Arg-tRNA and is catalyzed by ATE1, which helps in its recognition by PRT6 N-recognin for its ubiquitin-mediated proteolysis. MAPs—aminopeptidases; PCOs—plant cysteine oxidases; PTMs—post-translational modifications; ATE1—arginyl-transferases; PRT6—proteolysis 6 E3 ligases. The model structure of RAP2.3 was obtained from UniProt (https://www.uniprot.org/uniprot/P42736#structure accessed on 19 December 2021). The structure of Arg-tRNA^Arg^ and ATE1 is reprinted (adapted) with permission from [65].

Reports suggest the ubiquitin–proteasome pathway acts as part of the NO sensing mechanism in plants through the ERFs-VII group of TFs [15]. The group ERFs-VII are TFs that form a phylogenetic cluster in the large ERF TFs family which comprise a large number of elements specified in plants [58]. The stability of ERF-VII TFs is regulated by intracellular oxygen and NO levels through the N-end rule pathway (or the N-degron) [15]. This regulation was first studied under hypoxic (low oxygen) conditions [48]. It is noteworthy here that even under hypoxic conditions, various biochemical pathways, including the energy production pathways, lead to the production of reactive oxygen species (ROS) [59,60]. This process is similar to the one that occurs in mammalian cells under hypoxia, suggesting that ROS are produced in the modulation of hypoxic signaling [61]. This hypoxic signaling enables plants to sense the low environmental oxygen levels through the hypoxia-responsive transcription factors, such as ERF-VII TFs groups [62]. In flowering plants, this pathway was found to require NO in addition to oxygen [14]. This indicated that the N-end rule pathway is also an NO sensor in plants [15]. Thus, according to this pathway, in the presence of low oxygen and NO, ERFs-VII TFs are destabilized due to the cleavage of N-terminal methionine by endopeptidase or its cotranslational cleavage by aminopeptidases (MAPs), exposing the second cysteine residue (Cys2) of the ERF transcription factor for oxidation by plant cysteine oxidases (PCOs). The oxidation of Cys2 is further arginylated by arginine-tRNA (Arg-tRNA) and catalyzed by arginyl-transferase (ATE) [63,64], followed by recognition of the arginine destabilizing residue by N-recognins (PRT6; proteolysis six E3 ligase) and the ubiquitin proteasomal system which leads them to the 26S proteasome for degradation (Figure 1).

In a study, Arabidopsis proteome analysis depicted only 246 potential targets of the N-degron pathway [14]. This also indicates the specificity and the limitation of protein regulation by the N-degron mechanism, and particular biological responses. Some of the potential targets of the N-degron pathway, other than ERF-VII TFs, include VRN2 (vernalization 2) [66], PRC-2 (a subunit of the polycomb repressive complex) [49], and ZPR2 (little zipper 2 transcription factor) [50].

## 5. NO-Mediated PTMs Associated with Ubiquitin-Mediated Proteolysis

NO signal perception and transduction occur through NO-mediated post-translational modifications (PTM) of target proteins in plants. NO-mediated PTMs include S-nitrosylation, where NO reacts with the thiol group of cysteine (Cys) residue to form S-nitrosothiol; tyrosine nitration, the formation of 3-nitrotyrosine by peroxynitrite (NO-derived species); and metal nitrosylation, the binding of NO to transition metals of metalloproteins [12]. Recent studies suggest that the addition of NO to reactive Cys thiols, in the process of S-nitrosylation of proteins, may act as a key mechanism of NO signaling in plants [13,67].

Moreover, S-nitrosylation is a well-studied, essential regulatory PTM involved in plant immune responses [68]. Several other processes involve proteins that are potential targets for S-nitrosylation in Arabidopsis [69]. Proteins involved in plant immune response are well-studied targets of s-nitrosylation. These include NADPH oxidase [70,71], SALICYLIC ACID-BINDING PROTEIN 3 (SABP3) [72], NON-EXPRESSOR OF PATHOGENESIS-RELATED GENES 1 (NPR1) [73], TGACG-binding factor 1 (TGA1) [74], ASCORBATE PEROXIDASE 1 (APX1) [75], etc. Out of these, S-nitrosylation of APX1 and ABI5 (ABA INSENSITIVE 5) are reported to undergo degradation through the ubiquitin–proteasome pathway, while NPR1 is protected from ubiquitylation after S-nitrosylation [76,77,78]. However, soluble receptors in ABA signaling are targets of tyrosine nitration but are directed for proteasomal degradation in a similar manner.

**Figure 2:**
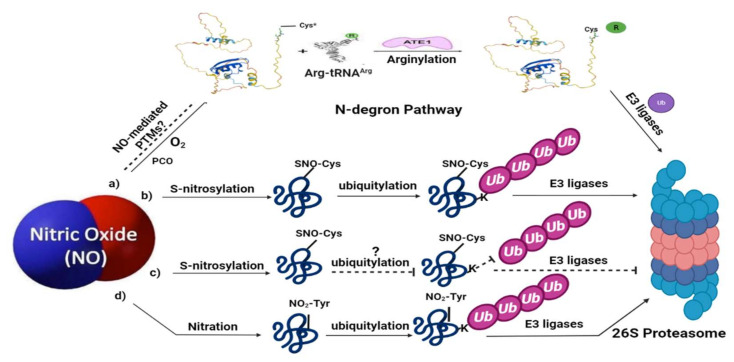
Nitric oxide signaling associated with ubiquitin-mediated proteolysis in plants: (**a**) represents the N-degron pathway (already described in detail in Figure 1); (**b**) under certain environmental conditions, NO can trigger ubiquitin-mediated proteasomal degradation of some proteins via *S*-nitrosylation, for example, APX1 and ABI5; (**c**) meanwhile, NO can also protect certain proteins by preventing their degradation via *S*-nitrosylation; (**d**) NO-mediated PTMs, such as tyrosine nitration, also leads to the proteolytic degradation via ubiquitin-mediated PTMs. Limited evidence is available for these pathways in plants; therefore, the dashed lines and question marks are used which represent further clarification of these signaling pathways in plants. SNO represents *S*-nitrosothiol; NO_2_-Tyr represents tyrosine nitration. The structure of Arg-tRNA^Arg^ and ATE1 is reprinted (adapted) with permission from [65].

Ascorbate peroxidases are key antioxidative enzymes, involved in scavenging H_2_O_2_ using ascorbate as an electron donor. The cytosolic isoform of ascorbate peroxidase, *apx1*, plays a key role in regulating H_2_O_2_ levels under abiotic stress conditions [59,79] and also constitutes an essential part of the control of redox metabolism during plant immune responses [80]. Recent reports have suggested that S-nitrosylation, nitration, and metal nitrosylation regulate APX1 activity during different cellular processes [81]. Under oxidative stress, S-nitrosylation of APX1 is suggested to enhance the enzymatic activity of APX1 in scavenging hydrogen peroxide [75]. Interestingly, a study discussed that S-nitrosylation of cAPX induces its ubiquitylation and degradation which acts as a signaling mechanism of programed cell death in tobacco cells [76]. This study reports that hydrogen peroxide induces programed cell death in tobacco cells through S-nitrosylation of cAPX which drives it to ubiquitin-mediated proteasomal degradation (as shown in Figure 2b). Moreover, the treatment of cells with an NO scavenger, cPTIO (2-4-carboxyphenyl-4,4,5,5-tetramethylimidazoline-1-oxyl-3-oxide), remarkably prevented ubiquitylation and degradation of cAPX, indicating that S-nitrosylation is a signal for ubiquitin-mediated protein degradation. Such a mechanism was found to be similar in animals where S-nitrosylation is a direct signal for ubiquitin-mediated protein degradation [82].

ABI5 (ABA INSENSITIVE 5) is a basic leucine zipper-type transcription factor, which is regulated by the stress hormone, abscisic acid (ABA). It is responsible for the ABA-induced growth arrest after germination. This effect is counteracted by NO during seed germination. It has been reported that in the presence of NO, S-nitrosylation of ABI5 occurs which is followed by its catalytic degradation through CULLIN4-based and KEEP ON GOING E3 ligases, thus promoting seed germination [78] (as shown in Figure 2b). ABA signaling also involves ligand binding by a PYRABACTIN RESISTANCE1/PYR1-LIKE/REGULATORY COMPONENT (PYR/PYL/RCR) family of soluble receptors and the interaction of this receptor-ligand complex with the PP2C family of PROTEIN PHOSPHATASES 2C [83,84]. This complex inhibits phosphatase activity, enabling the ABA-activated SUCROSE NON-FERMENTING1 KINASES RELATED SUBFAMILY 2 (SnRK2) protein kinase family () to remain phosphorylated and initiate the phosphorylation of ABA-responsive TFs. Since NO exerts a negative effect on ABA signaling [85] it is reported that NO mediates the tyrosine nitration of ABA receptors of the PYR/PYL/RCAR family [86]. Tyrosine nitration of these ABA receptors reduces their activity and they are polyubiquitylated for proteasome-mediated degradation [86] (as shown in Figure 2d), thus limiting the phosphorylation of ABA-responsive TFs and negatively affecting the ABA signaling response.

NPR1 (NON-EXPRESSOR OF PATHOGENESIS-RELATED GENES 1) is a key player in the establishment of salicylic acid (SA)-induced systemic acquired resistance [46] and induced systemic response (ISR) in response to pathogen challenge in plants [87,88]. NPR1 exists as an oligomer in the cytoplasm under non-stress conditions. However, under stress conditions, an oxidative burst leads to its monomerization, nuclear localization, and thereafter, the expression of pathogenesis-related (PR) genes [89]. This oligomer-monomer switch involves S-nitrosylation of NPR1 on cysteine 156, which translocates it to the nucleus and allows further interaction with the TGA TF (another target for S-nitrosylation) for the transcription of PR genes [74,89]. However, NPR1 is ubiquitinylated and undergoes proteasomal degradation in the absence of a pathogen. It has been reported that NPR1 undergoes stepwise ubiquitylation by CULLIN3-RING E3 ligase and E4 ligase, respectively, for catalyzing polyubiquitylation [30,90]. A study by Ding et al. [77] suggests that where ABA promotes the ubiquitin-mediated proteasomal degradation of NPR1, S-nitrosylation could protect it (as shown in Figure 2c).

## 6. NO-Mediated PTMs Regulate the Components of Ubiquitin-Mediated Proteolysis

NO-mediated PTMs can not only modify the target protein but can also modify the components of the ubiquitylation machinery, including E1–E3 enzymes and the proteasome for promoting or inhibiting degradation of the target protein. This mechanism has been well studied in animals [91,92,93]. In plants, however, more studies are required to project a clear picture of this mechanism. So far, a few studies have reported S-nitrosylation of the E3 ligase complex that amplifies its effect on the target protein for degradation. In Arabidopsis, it includes a ubiquitin-activating enzyme, an alpha-subunit, and a regulatory subunit of 26S proteasome as targets of S-nitrosylation [94]. Another example is the TRANSPORT INHIBITOR RESPONSE 1/AUXIN SIGNALING F-BOX (TIR1/AFB) protein which is a substrate receptor subunit of the E3-ubiquitin ligase complex, SCF^TIR1/AFB^. This complex is responsible for the degradation of AUXIN/INDOLE-3-ACETIC ACID (Aux/IAA) repressors to induce auxin-regulated responses. S-nitrosylation of the TIR1 auxin receptor at Cys140 enhances the TIR1-Aux/IAA interaction, and thus facilitates its ubiquitylation and degradation while promoting the auxin-regulated response [95].

Another interesting candidate with immediate links with the ubiquitin pathway is CELL DIVISION CONTROL 48, CDC48 (p97). In yeast, plants, and animals, CDC48, a cell division control protein also known as p97 or VALOSIN-CONTAINING PROTEIN (VCP), is present, which belongs to the evolutionary conserved ATPase of the AAA family. This abundant protein plays a major regulatory role in a broad array of cellular processes, particularly the proteasome-dependent degradation of ubiquitylated proteins, redox regulation, and subcellular trafficking [96,97]. Of particular interest is its ‘segregase’ activity which involves segregating ubiquitylated substrates from unmodified partners. S-nitrosylation of CDC48 at conserved cysteine residues inhibits its ATPase activity [98] during the host–pathogen interaction between *Nicotiana tabacum* and *Phytopthora cryptogea*. During their interaction, the pathogen, *P. cryptogea*, produces several avirulence factors, known as elicitins. One of the elicitins produced by *P. cryptogea* is cryptogein, which induces S-nitrosylation of NtCDC48 at Cys526 in *Nicotiana* cells, compromising its immunity [99,100]. An extension of this study suggests the regulation of the ubiquitin–proteasome activity of CDC48 in response to the cryptogein [101]. Table 1 summarizes the association between NO-mediated PTMs and the process of ubiquitylation on target proteins.

## 7. Conclusions

The fundamental role of NO in plant biology has always been fascinating because of its involvement in almost all plant processes, modulating phytohormonal signaling, and environmental acclimation processes. Its involvement in the ubiquitin-mediated proteasomal degradation is yet another interesting discovery that unfolds its regulatory role on other proteins involved in key signaling pathways. The mechanism of NO signal perception and transduction through NO-mediated PTMs has been one of the most-studied signaling mechanisms in plants. In the presence of NO, some regulatory proteins get modified through these NO-mediated PTMs or the N-degron pathway which leads them to degradation via ubiquitylation. NO-mediated PTMs may also target the components of ubiquitylation machinery for inducing the degradation of proteins. Recently, NO sensing through the N-degron pathway has gained much attention due to its direct NO sensing mechanism which directs the target proteins to ubiquitin-mediated proteasomal degradation. Moreover, as in the case of NPR1, NO-mediated PTMs may be involved in the protection of regulatory proteins from degradation due to their key role in plant immune response or other environmental conditions, which further associates NO signaling to the ubiquitylation process. Hence, there seems to be a clear link between NO sensing and the ubiquitylation of proteins for maintaining the cellular homeostasis in plants for survival under changing environmental conditions. Further experimental research needs to be done to clearly elucidate the link between NO-mediated PTMs and ubiquitylation.

## Figures and Tables

**Table 1:** Ubiquitin-mediated proteasomal degradation of proteins associated with NO-mediated PTMs in plants.

Targets for S-Nitrosylation	NO-Mediated PTMs	Target for Ubiquitylation	Ubiquitin–Proteasomal Machinery	Purpose	References
**ABI5**	S-nitrosylation at Cys153	ABI5	CULLIN4-based and KEEP ON GOING E3 ligases	Germination and early seed development	[78]
**NPR1**	S-nitrosylation at Cys156	NPR1	ABA-mediated degradation through CULLIN3-RING E3 ligase and E4 ligase	Protection from ABA-mediated proteasomal degradation.	[89]
**ABA receptors of the PYR/PYL/RCAR family**	Tyrosine nitration	PYR1	Polyubiquitinylation for degradation.	Reduce the activity of ABA receptors and hence limit ABA response.	[86]
**cAPX1**	S-nitrosylation at Cys32	cAPX1	Ubiquitylation for degradation.	H_2_O_2_ -mediated programed cell death	[75,76]
**TIR1**	S-nitrosylation at Cys140 and Cys480	Aux/IAA repressors	E3-ubiquitin ligase complex, SCF^TIR1/AFB^	Degradation of Aux/IAA repressors to induce auxin-regulated responses.	[95]
**CDC48**	S-nitrosylation at Cys526	CDC48	Itself has ubiquitin–proteasome activity	Compromised immunity against *P. cryptogea*	[99,101]
