# Correction: Pande et al. Nitric Oxide Signaling and Its Association with Ubiquitin-Mediated Proteasomal Degradation in Plants. *Int. J. Mol. Sci.* 2022, *23*, 1657

**DOI:** 10.3390/ijms23105628

**Published:** 2022-05-18

**Authors:** Anjali Pande, Bong-Gyu Mun, Murtaza Khan, Waqas Rahim, Da-Sol Lee, Geun-Mo Lee, Tiba Nazar Ibrahim Al Azawi, Adil Hussain, Byung-Wook Yun

**Affiliations:** 1 Laboratory of Plant Molecular Pathology and Functional Genomics, Department of Plant Biosciences, School of Applied Biosciences, College of Agriculture & Life Science, Kyungpook National University, Daegu 41944, Korea; mun0301@naver.com (B.-G.M.); murtazakhan.bio@gmail.com (M.K.); waqasrahim999@yahoo.com (W.R.); giftanna@naver.com (D.-S.L.); looxia@naver.com (G.-M.L.); redflower660@yahoo.com (T.N.I.A.A.); 2 Laboratory of Cell Biology, Department of Entomology, Abdul Wali Khan University, Mardan 23200, Khyber Pakhtunkhwa, Pakistan; adilhussain@awkum.edu.pk

The authors wish to make the following corrections to the original publication [1]. In the original publication, there was a mistake in the legend for Figures 1 and 2. In Figures 1 and 2, the structure of Arg-tRNA^Arg^ and ATE1 overlooked the citation of the original work from where it was adapted [x]. The missed citation [x] is, therefore, clarified in the corrected legends (below) as reference [2]. The correct legends for Figure 1 and Figure 2 appear below.

The authors apologize for any inconvenience caused and state that the scientific conclusions are unaffected. This correction was approved by the Academic Editor. The original publication has also been updated.

## Figures and Tables

**Figure 1:**
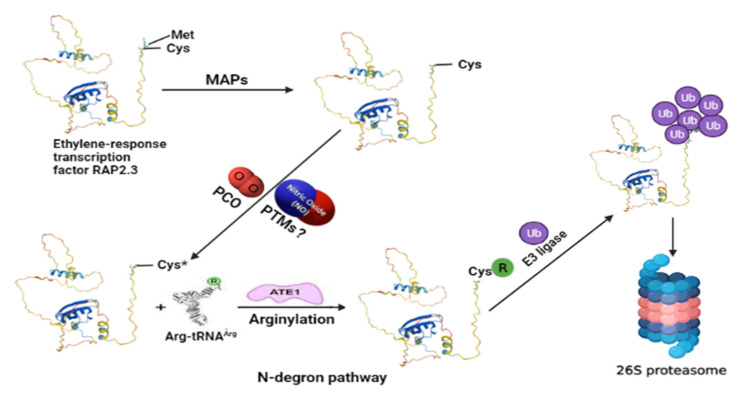
An exemplified model for understanding the N-degron pathway involving ubiquitin proteasomal degradation of ERF-VII (for example, RELATED TO AP2 3, RAP2.3) TF as a part of the NO sensing mechanism in plants. The N-terminal methionine is cleaved by MAPs exposing the second residue, cysteine (Cys). Cysteine gets oxidized by the action of PCOs. In this step, the role of NO is also reported, so possible NO-mediated oxidation of cysteine residue via PTMs still needs to be explored. The oxidized cysteine (Cys*) of the target protein then undergoes arginylation by Arg-tRNA and is catalyzed by ATE1, which helps in its recognition by PRT6 N-recognin for its ubiquitin-mediated proteolysis. MAPs—aminopeptidases; PCOs—plant cysteine oxidases; PTMs—post-translational modifications; ATE1—arginyl-transferases; PRT6—proteolysis 6 E3 ligases. The model structure of RAP2.3 was obtained from UniProt (https://www.uniprot.org/uniprot/P42736#structure accessed on 19 December 2021). The structure of Arg-tRNA^Arg^ and ATE1 is reprinted (adapted) with permission from [2].

**Figure 2:**
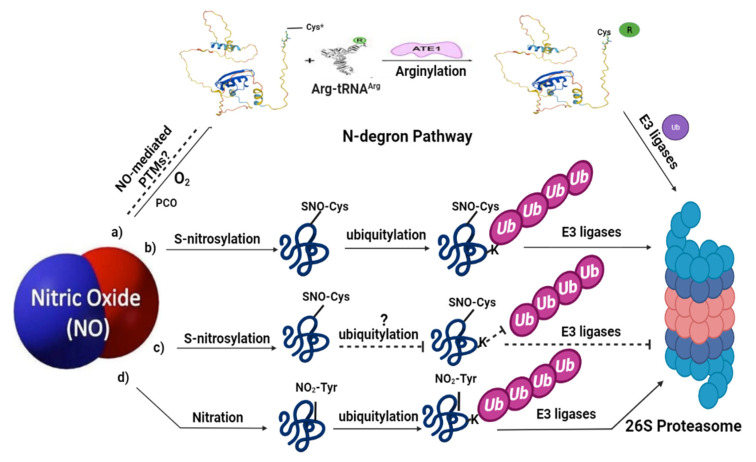
Nitric oxide signaling associated with ubiquitin-mediated proteolysis in plants: (**a**) represents the N-degron pathway (already described in detail in Figure 1); (**b**) under certain environmental conditions, NO can trigger ubiquitin-mediated proteasomal degradation of some proteins via *S*-nitrosylation, for example, APX1 and ABI5; (**c**) meanwhile, NO can also protect certain proteins by preventing their degradation via *S*-nitrosylation; (**d**) NO-mediated PTMs, such as tyrosine nitration, also leads to the proteolytic degradation via ubiquitin-mediated PTMs. Limited evidence is available for these pathways in plants; therefore, the dashed lines and question marks are used which represent further clarification of these signaling pathways in plants. SNO represents *S*-nitrosothiol; NO_2_-Tyr represents tyrosine nitration. The structure of Arg-tRNA^Arg^ and ATE1 is reprinted (adapted) with permission from [2].
